# The absence of reproductive isolation between non-sister and deeply diverged mitochondrial lineages of the black-throated tit (*Aegithalos concinnus*) revealed by a multilocus genetic analysis in a contact zone

**DOI:** 10.1186/s12862-017-1114-9

**Published:** 2017-12-20

**Authors:** Chuanyin Dai, Yan Hao, Yong He, Fumin Lei

**Affiliations:** 1 School of Chemistry and Life Sciences, Guizhou Normal College, Gaoxin Road 115, Guiyang, Guizhou 550018 China; 2 Key Laboratory of Zoological Systematics and Evolution, Institute of Zoology, Chinese Academy of Sciences, Beichen Xi Road 1, Beijing, 100101 China; 3 University of Chinese Academy of Sciences, Beijing, 100049 China

**Keywords:** Black-throated tit, Speciation in reverse, Divergence time, Reproductive isolation, Male-biased dispersal

## Abstract

**Background:**

A deep divergence of mitochondrial DNA is common in species delimitated by morphological traits. Several hypotheses can explain such variations, such as cryptic species, introgression, allopatric divergence and ancestral lineage. The black-throated tit harbors several deeply divergent mitochondrial lineages. Two lineages with 5% divergence, but having a high level of gene flow, have been detected in its subspecies, *A. C. concinnus* and *A. c. talifuenses*. In this study, we conducted a genetic analysis at a contact zone of these two lineages to identify whether these lineages either reflect a high level of intraspecific variation in mitochondrial loci or represent incipient speciation. Mitochondrial ND2 and 11 microsatellite loci were used to conduct phylogenetic and population structure analyses.

**Results:**

ND2 haplotypes actually diverged into two groups within subspecies *A. c. talifuenses*; however, they formed a non-sister relationship when including all available GenBank ND2 sequences. Analyses of microsatellite data indicated no existing population structure and showed a pattern of isolation by distance. Individuals sampled at the contact zone were almost identified as F2 hybrids.

**Conclusions:**

Isolation for 2.4 Ma, as suggested by a previous study, appeared to be insufficient to develop robust reproductive barriers. Reproductive barriers were weak, or even absent between the divergent lineages, highlighting that incipient speciation was unlikely to be the case. Considering the results from previous studies, the divergent lineages may be better explained by secondary contact after allopatric isolation because of Pleistocene climate changes, but other hypotheses cannot be definitively ruled out because of the lack of representative samples from the other distribution region and its relatives. Considering that divergence in morphology was hardly observed and the particular split in genetics, the two subspecies might be better merged. The current findings also highlight the likely contribution of male-biased dispersal to male-biased gene flow among mitochondrial lineages; more efforts are needed to illustrate the evolutionary history of the black-throated tit.

**Electronic supplementary material:**

The online version of this article (doi: 10.1186/s12862-017-1114-9) contains supplementary material, which is available to authorized users.

## Background

A growing number of population genetics and phylogeographic studies have noted that a large number of species are genetically structured [1, 2], particularly with respect to mitochondrial DNA, because of their fast evolutionary rate and small effective size [3]. The level of intraspecific divergence in mitochondrial loci that is characterized by some species can reach the level of interspecies, such as the divergence occurring in several birds, e.g., the gray-cheeked fulvetta (*Alcippe morrisonia*) [4, 5], the common raven (*Corvus corax*) [6, 7], and the common redstart (*Phoenicurus phoenicurus*) [1]. For the above mentioned species, the mitochondrial lineages have diverged to the extent of at least 4%. Several hypotheses can explain such high variation, such as cryptic species, introgression, allopatric divergence and ancestral lineage [8, 9]; many factors can contribute to such variation within a species, such as historical geographic events, paleoclimatic changes, current geographic isolation and attributes of organisms. In some cases, a deeply diverged lineage in avian species was actually a cryptic species without any distinct morphological changes or the lineage reflected a speciation event in the species’ evolutionary history, with paralleling divergence in phenotypic characteristics [10–12]. However, the use of nuclear DNA to infer a species’ population structure may show a discordant pattern compared with that of mitochondrial DNA [13]. Such cyto-nuclear discordance has been the subject of broad debate in past decades [13–15] and, as a result, has highlighted that more efforts and multiple criteria are needed when the delimitation of a species is concluded [16–18].

The black-throated tit is a small and abundant passerine bird with a broadly continuous distribution in Pakistan, Nepal, Bhutan, Indian, Myanmar, Thailand, Laos, Vietnam and mainland China (http://www.hbw.com). Isolated populations inhabit southern Laos, central Vietnam, Cambodia and the island of Taiwan [19]. This species is highly variable both in phenotype and mitochondrial diversity. Currently, at least six subspecies are recognized based on morphological traits. All subspecies have a similar pattern and arrangement of plumage, for example, a black throat and a black “bandit mask” around the eye. However, most of the subspecies feature different colors in other parts of their bodies, such as their crowns, bellies and flanks, and the color and width of their postocular lines vary as well. Recently, genetic analyses of four subspecies revealed that mitochondrial variation was notable not only between subspecies but also within certain subspecies [20, 21]. The marked geographical variation in morphology, combined with the substantial genetic split, has been suspected to be representative of several reproductively isolated species [20].

Mitochondrial variation was previously detected in three monophyletic lineages, in which the genetic distance was as high as 5% between the most divergent lineages in subspecies *A. C. concinnus* and *A. c. talifuensis* [22]. However, the use of several nuclear sequence loci has failed to show a concordant pattern of genetic structure. Very weak population structures have been observed in nuclear sequence loci, and coalescent analyses have detected high levels of gene flow among mitochondrial-defined lineages, even between allopatric populations [23]. As such, the cyto-nuclear discordance was explained by male-biased gene flow and excluded the possibility of incomplete lineage sorting in nuclear loci. These findings indicated that complete reproductive isolation was not the case between these divergent lineages. However, whether the detected gene flows were restricted by incomplete reproductive barriers requires further consideration. Namely, the divergent lineages may represent either a nascent species or simply a reflection of a high level of genetic diversity within a species.

When reproductive isolation is incomplete, genetically distinct populations or lineages can come into contact and interbreed, producing individuals genetically mixed in the hybrid zones, which have long been regarded as natural laboratories for understanding the process of speciation [24, 25]. Hybrid zones may be primary, which occurs between sympatric and parapatric populations mostly influenced by different environmental selection factors, or they may be secondary, which occurs between formerly allopatric populations that interact [24–26]. The underlying structure of a hybrid zone is determined by the interaction of several factors, such as the genetic characteristics of the hybrid individuals, natural selection, modes of gene flow and landscape properties [24, 27, 28]. Nevertheless, the fitness of offspring with mixed ancestry can predict the type of hybrid zone [29]. Reduced fitness of hybrids caused by endogenous or exogenous selection will form a bimodal zone and higher fitness relative to parental genotypes bounded by a superior modal zone or even hybrid species, whereas equal fitness between hybrids and parentals will result in a unimodal zone owing to random mating among hybrids and between hybrids and parentals [28, 30].

Thus, studies of hybrid zones are paramount in assessing taxonomic status and systematics, as well as for providing insight into the mechanisms that result in reproductive isolation and speciation. For example, a genetic analysis conducted at the contact zone between two subspecies of the ocellated lizard (*Lacerta lepida*) in southeastern Iberia showed a narrow and steep hybrid zone, highlighting the different evolutionary trajectories and independent species identity for these two subspecies [31]. Analyses of two lineages of the mule deer (*Odocoileus hemionus*), however, revealed a hybrid swarm at their hybrid zone, indicating a lack of prezygotic and postzygotic barriers despite the dramatic morphological, ecological and genetic differences between the two lineages [29].

Because such relevant information can be provided by analyzing the genetic characteristics of the contact zone, we sampled individuals from a contact region between two deeply divergent mitochondrial lineages harbored in the two subspecies of the black-throated tit: *A. C. concinnus* and *A. c. talifuensis*, which have similar plumage, with the former exhibiting a brighter red color [32]. A previous study indicated that the divergent lineages occurred in neighboring areas near the administrative border between the Yunnan and Guizhou Provinces [22]. We amplified one mitochondrial and eleven microsatellite loci to infer the degree of reproductive isolation by reconstructing the population genetic structure and to evaluate the modality of its hybrid zone. As a high level of gene flow has been observed, if these two lineages were at a certain stage of the speciation process, reproductive barriers should exist and hybridization would create a bimodal zone where parental populations are the dominant types. Otherwise, lineages would form a unimodal zone if reproductive barriers were weak, or even absent, and thus, random interbreeding would be the cause. In this case, the divergence between lineages should be better attributed to the high level of differentiation (neutral or adaptive) within a species and may reflect a historical artifact [8, 9].

Additionally, we tested the relationship between divergence time and the level of reproductive isolation in the black-throated tit. A key assumption in evolutionary biology is that reproductive barriers between lineages would gradually increase with genetic divergence and thereby would accumulate with divergence time [33, 34]. While the literature suggests that species exhibiting nearly complete reproductive isolation may need more than 7 Ma under natural conditions [35, 36], recent studies conducted in anurans have indicated that the formation of a hybrid zone with restricted gene flow may require more than 3 Ma for allopatric distribution [37–40]. Given that a previous study [22] estimated that lineages were isolated by Pleistocene climatic change approximately 2.4 Ma ago, we expected the presence of weak reproductive barriers between the divergent lineages and that these lineages would form a wider and unimodal hybrid zone.

## Methods

### Sampling

**Fig. 1:**
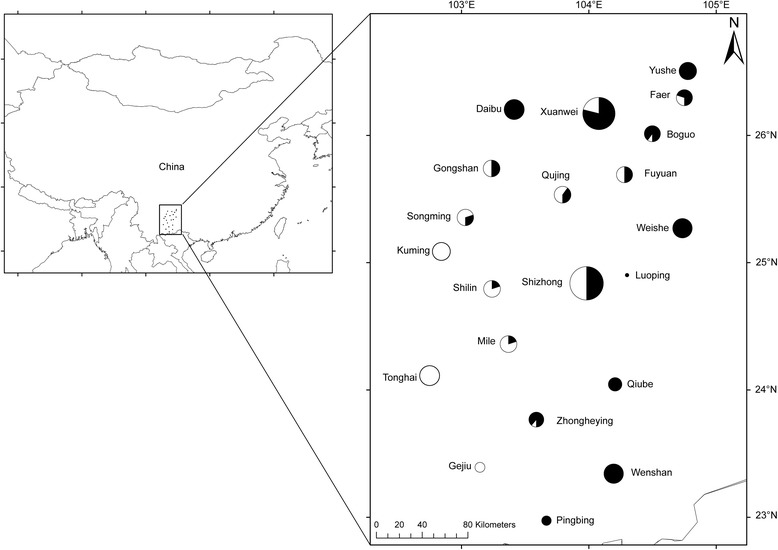
Sampling sites for the current study and the geographic distribution of the mitochondrial ND2 haplotypes. White indicates haplotypes belonging to lineage A, whereas black refers to those of lineage B-C

We collected specimens from neighboring areas near the administrative border between the Yunnan and Guizhou Provinces (Fig. 1), which have been identified as a potential contact zone between divergent lineages harbored in *A. C. concinnus* and *A. c. talifuensis* [22]. Field work was carried out from January to February and from November to December 2015. Birds were captured with mist nets, and the recorded voice of a population of the black-throated tit from Taiwan was used to find and attract the targeted species. Blood or muscle was collected and stored in pure ethanol at room temperature. The number of specimens collected was approved by the ethics committee of Guizhou Normal College. The collection was also carried out with permits from the local forestry administrations at each collection site. In total, 205 individuals from 21 localities were collected for the present study (Additional file 1: Table S1); the voucher specimens were deposited under numbers GZ H1–65 and GZ H67–206 at Guizhou Normal College.

### Molecular methods

DNA was extracted with the DNeasy Tissue & Cell kit (Aidlab, China) according to the manufacturer’s protocols. The sequence of the ND2 coding gene was amplified with the primers H6313 and L5219 following the methods for polymerase chain reaction (PCR) outlined in [22]. ND2 PCR products were sequenced in both strands using the amplifying primers. Sequencing services were provided by the BGI Company. Sequences were assembled using the Seqman algorithm implemented in DNASTAR and visually compared with the original chromatograms to avoid reading errors. Additionally, we downloaded all available GenBank ND2 sequences for the black-throated tit. For comparison, we further downloaded all available GenBank Cyt B sequences. The total number of downloaded sequences was 212 for ND2 and 223 for Cyt B. These sequences varied in length, and both covered four subspecies. Sequences with ambiguous bases were excluded from the following analyses. We genotyped all the collected individuals at 11 microsatellite loci: Ase18, Ase37, Escu6, Man13, Pca3, PmaD22 [41], TG01040, TG03031, TG04004, TG04041 and TG01147 [42]. Protocols for PCR were followed [43], and amplicons were sized using capillary electrophoresis with an ABI 3730xl DNA analyzer by a service provider (Kunming TsingKe BioTech). Alleles were scored using GeneMapper software version 4.0 (Applied Biosystems Inc.). To ensure the quality of genotyping, all electropherograms were visually checked.

### Mitochondrial data analyses

Sequences of ND2 and Cyt B were aligned using the ClustalW algorithm implemented in MEGA 6 [44]. For ND2, as the downloaded GenBank sequences varied in length, we trimmed the sequences generated in this study to match the downloaded sequences. As a result, the lengths of the sequences used for analysis were saved as 981 and 734 bp, respectively. One sequence from *A. C. manipurensis* was included in the latter. The number of segregating sites, haplotype diversity and nucleotide diversity of our sampled individuals, downloaded sequences and both (combined data) were estimated using DnaSP 5.0 [45]. McDonald & Kreitman’s test was used to examine the selective neutrality of the ND2 fragments [46]. For Cyt B, we saved the data with a length of 829 bp.

For ND2, haplotypes with different lengths (981 and 734) for the combined data were used to reconstruct the phylogenetic relationships using maximum likelihood (ML) and Bayesian methods (BI). ML and BI were also used for constructing the phylogeny of Cyt B haplotypes. We used GenBank sequences of the long-tailed tit (*Aegithalos caudatus*) as outgroups. Substitution models for data sets were selected using jModelTest 2.1.7 based on Akaike’s information criterion [47]. GTR + I was suggested as the best-fit model for both ND2 and Cyt B. ML trees were constructed using the online version of PhyML 3.0 (http://www.atgc-montpellier.fr/phyml/). We also applied the algorithm of automatic model selection to construct the phylogeny. The robustness of the phylogeny in the ML tree was tested by a bootstrap test with 100 resamplings. Bayesian inference (BI) was conducted using MrBayes 3.1.2 [48]. Four Metropolis-coupled Markov chain Monte Carlo algorithms (one cold and three heated) were run for 10 million iterations, with trees sampled every 1000 iterations. The first 3 million iterations (2500 trees) were discarded as a burn-in period; then, the remaining sampled iterations were used to estimate the posterior probabilities. Two independent Bayesian runs, initiated from random starting trees, were performed, and the log-likelihood values, posterior probabilities and average deviations of split frequencies were used to confirm the convergence of the chains; for example, convergence was confirmed for average standard deviations below 0.01 and potential scale reduction factors (PSRFs) close to 1.

### Microsatellite data analyses

Micro-Checker [49] was used to detect genotyping errors as the results of null alleles, stuttering and allelic dropout. We used GenAlEx 6.5 [50] software to calculate the number of alleles, the expected heterozygosity (H_E_) and observed heterozygosity (H_O_) for each locus and population. Allelic richness was standardized for sample size by rarefaction in *Fstat* 2.9.3 [51]. We also tested departures from the Hardy-Weinberg equilibrium (HWE) per locus and population and deviation from the linkage equilibrium (LD) for all pairwise locus combinations. Significance was adjusted for multiple tests using Bonferroni corrections. These calculations were performed using Genepop 4.0 online (www.genepop.curtin.edu.au/index.html).

To test for geographic genetic population structure, analyses of molecular variance (AMOVA) with 10,000 permutations were conducted with Arlequin version 3.1 software [52, 53]. Sampled populations were grouped into two geographical lineages based on the results of ND2 phylogenetic analyses and were assessed according to the degree of differentiation between lineages (Φ_CT_), between sampled populations within lineages (Φ_SC_) and between all sampled populations (Φ_ST_). We further tested for isolation by distance among all localities by regressing geographical distance against *F*_ST_. Linear Euclidean distances (in kilometers) between samples were calculated from their metric easting and northing coordinates using the program Geographic Distance Matrix Generator (version 1.2.1) (Ersts Internet). Mantel tests with 10,000 permutations were performed with IBDWS version 3.14 (www.ibdws.sdsu.edu/) [54] to assess the statistical significances in correlation between genetic and geographical distances.

We used the individual-based Bayesian clustering method in the program STRUCTURE version 2.3.4 [55, 56] to investigate the overall population structure based on microsatellite allele frequencies. We performed ten runs at each hypothesized number of subpopulations (K). Runs of 200,000 permutations after 100,000 MCMC burn-ins were conducted from K = 1 to 21 using the admixture model, with allele frequencies assumed to be correlated [56]. All other parameters were retained as defaults. To select the most likely K, we followed the recommendations for interpreting results from [57], as well as by using ln(Pr(X|K) values to identify the k for which Pr(K = k) is highest. These results were computed and visualized in the online interface CLUMPAK [58].

We performed analysis with NEWHYBRIDS v.1.1 [59] to assign the sampled black-throated tits to six genotypic classes based on microsatellite data set. The method computes the Bayesian posterior probability of assignment of each individual to these different genotypic classes (parental, F1, F2, and backcrosses). We used default genotypic classes with no prior information on allelic frequencies and included Jeffreys and uniform priors for both θ and π. Runs were repeated 5 times with 100,000 sweeps of burn-in and 1,000,000 sweeps of data collection.

## Results

### Mitochondrial data

A total of 204 specimens were sequenced successfully for the mitochondrial ND2 gene. These newly generated data, with a length of 734 bp, contained 66 polymorphic sites—59 of which were parsimony informative—and defined 27 haplotypes, whereas the data with a length of 981 bp had 84 polymorphic sites—75 of which were parsimony informative—and identified 32 haplotypes. Sequences available in GenBank had 64 haplotypes based on 734 bp of 208 sequences, while 75 haplotypes were found based on 981 bp of 207 sequences. The combined data with a length of 734 bp defined 84 haplotypes, while 99 haplotypes were found for the combined data with a length of 981 bp (Table 1). These results revealed that the majority of the haplotypes identified in the current study were different from those previously defined, as only 7 and 8 haplotypes were shared, respectively. The result of the McDonald & Kreitman’s test was not significant, providing no evidence for major deviations from selective neutrality (Fisher’s exact test: *P* = 0.18).

**Table 1:** Summary of the genetic diversity of the ND2 sequences used in this study

Data resources	Length	Number of sequences	Polymorphic sites	Number of haplotypes
Combined	981	411	159	99
Combined	734	412	106	84
This study	981	204	84	32
This study	734	204	66	27
GenBank	981	207	146	75
GenBank	734	208	94	64

**Fig. 2:**
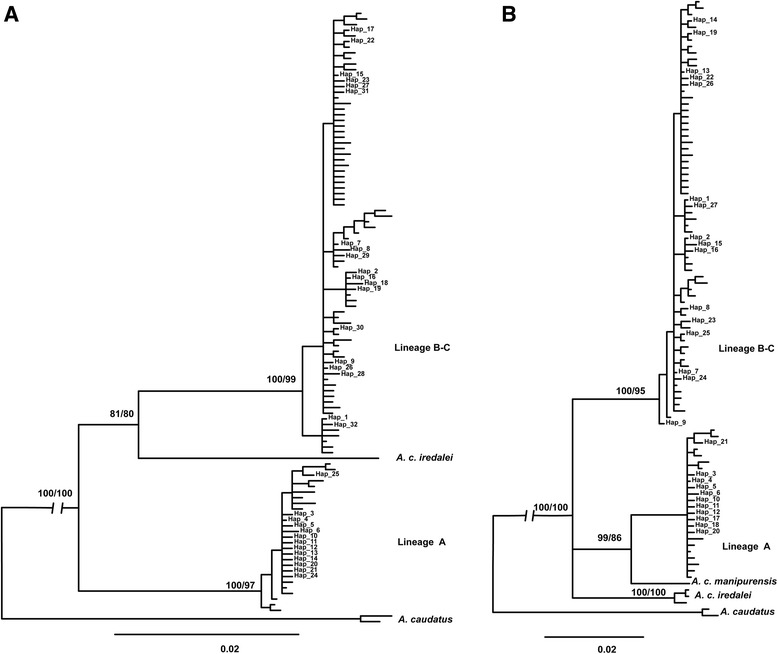
Phylogenetic trees based on the partial sequences of ND2 from this study and GenBank. Numbers indicated in the nodes are the support values based on ML and BI. Haplotypes belonging to lineages A and B-C with names are from the current study. Phylogeny A was constructed with 981 bp, whereas B was reconstructed with 734 bp

Major phylogenetic splits for the ND2 haplotypes identified in this study were concordant based on the analyses of different methods and sizes of sequences. Phylogenetic analyses showed that the haplotypes belonged to two deeply divergent lineages. This divergence was in agreement with the previous finding (A and B-C in [22]). Sympatry of deeply divergent lineages was observed at 11 locations, whereas 10 populations were dominated by one of the pure mitochondrial lineages. Based on phylogeny, we found that the divergence occurred within subspecies *A. c. talifuensis*, as samples used in this study were *A. c. talifuensis* based on morphology. Surprisingly, including the sequences from other subspecies revealed a non-sister relationship for the two targeted lineages despite their parapatric distribution. Based on the phylogeny constructed by the 981 bp of ND2 sequences, *A. C. iredalei* was the sister to lineage B-C (Node support was high in the ML tree), whereas lineage A was the sister to lineage B-C and *A. C. iredalei* (Fig. 2B). However, significant differences in the phylogenetic relationship were observed when the sequence of *A. C. manipurensis* was used. Lineage A was closely related to *manipurensis* (Node supports were high in the BI and ML trees), and a paraphyletic relationship was observed for lineage B-C, *A. C. iredalei* and lineage A-*manipurensis* (Fig. 2A)*.*

**Fig. 3:**
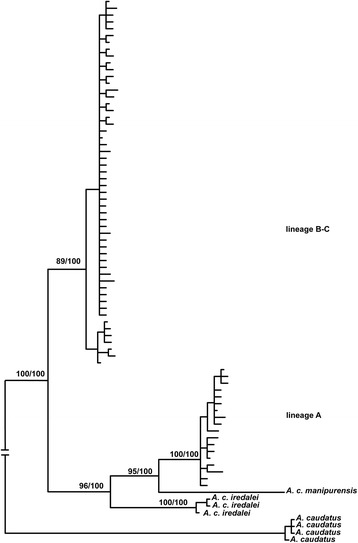
Phylogeny reconstructed with the available GenBank sequences of Cyt B. Node numbers refer to the support values of ML and BI

The phylogeny using the Cyt b haplotypes provided the same information concerning the phylogenetic relationship between lineages A and B-C identified in [22], highlighting its non-sister relationship. Lineage A was closely related to *manipurensis,* with *iredalei* as its sister group. Lineage B-C was the sister group to all three lineages. Most importantly, all nodes had high support in both the BI and ML trees (Fig. 3).

### Microsatellite data

Examination of the data with a micro-checker showed no evidence of null alleles, stuttering or allelic dropout. Among the individuals scored for the 11 microsatellite loci, significant deviations from HWE were detected in 4 of the 231 cases. The departures were also detected in 3 loci (Ase37, Escu6 and Pca3) and 4 sampled populations. No linkage disequilibrium was detected after correcting for multiple tests. A total of 126 alleles were found for 11 loci in our sampled populations. The mean number of alleles per locus was 9.719±0.249 SEM (range: 4 to 37 alleles). Allelic richness (Ar) varied across sites (1.537–3.718). H_O_ and H_E_ ranged from 0.251 to 0.892 and from 0.245 to 0.865, respectively (Table 2).

**Table 2:** Summary of the microsatellite loci of the black-throated tit in the contact zone

Locus	Sample size	Number of alleles	H_O_	H_E_	A_R_
Ase18	205	12	0.712	0.712	2.802
Ase37	202	18	0.757	0.784	2.986
Escu6	205	19	0.663	0.764	2.929
Man13	205	4	0.251	0.263	1.537
Pca3	203	6	0.541	0.59	2.261
PmaD22	204	37	0.892	0.926	3.718
TG01040	205	7	0.601	0.576	2.291
TG01147	205	4	0.563	0.535	2.110
TG03031	205	5	0.637	0.601	2.214
TG04004	204	9	0.471	0.449	1.999
TG04041	203	5	0.302	0.343	1.670

**Fig. 4:**
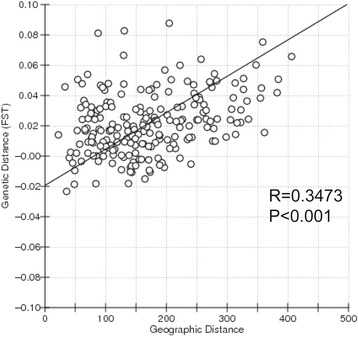
Analyses of isolation by distance indicating that divergence among the sampled populations can be explained by geographic distance

Analyses of molecular variance based on *F*_ST_ showed that most genetic variation (98.39%) occurred within sampled populations. Genetic variation among sampled populations within groups was only 1.59%, with that among groups accounting for only 0.02% of the total (Table 3). A significantly positive relationship between genetic distance and geographical distance matrices was detected across our sampled populations (*R* = 0.3473, *P* < 0.001), indicating that the structuring of sampled populations exhibited a pattern of isolation by distance (Fig. 4).

**Table 3:** Results of AMOVA

Source of variation	d.f.	Sum of squares	Variance components	Percentage of variation	Φ Statistics	*P*-value
Among groups	1	4.224	0.00069	0.02	Φ_CT_ = 0.00021	0.38612
Among populations with groups	30	117.852	0.05277	1.59	Φ_SC_ = 0.01591	0.0000
Within populations	378	1233.463	3.26313	98.39	Φ_ST_ = 0.01612	0.0000
Total	409	1355.539	3.31659

**Fig. 5:**
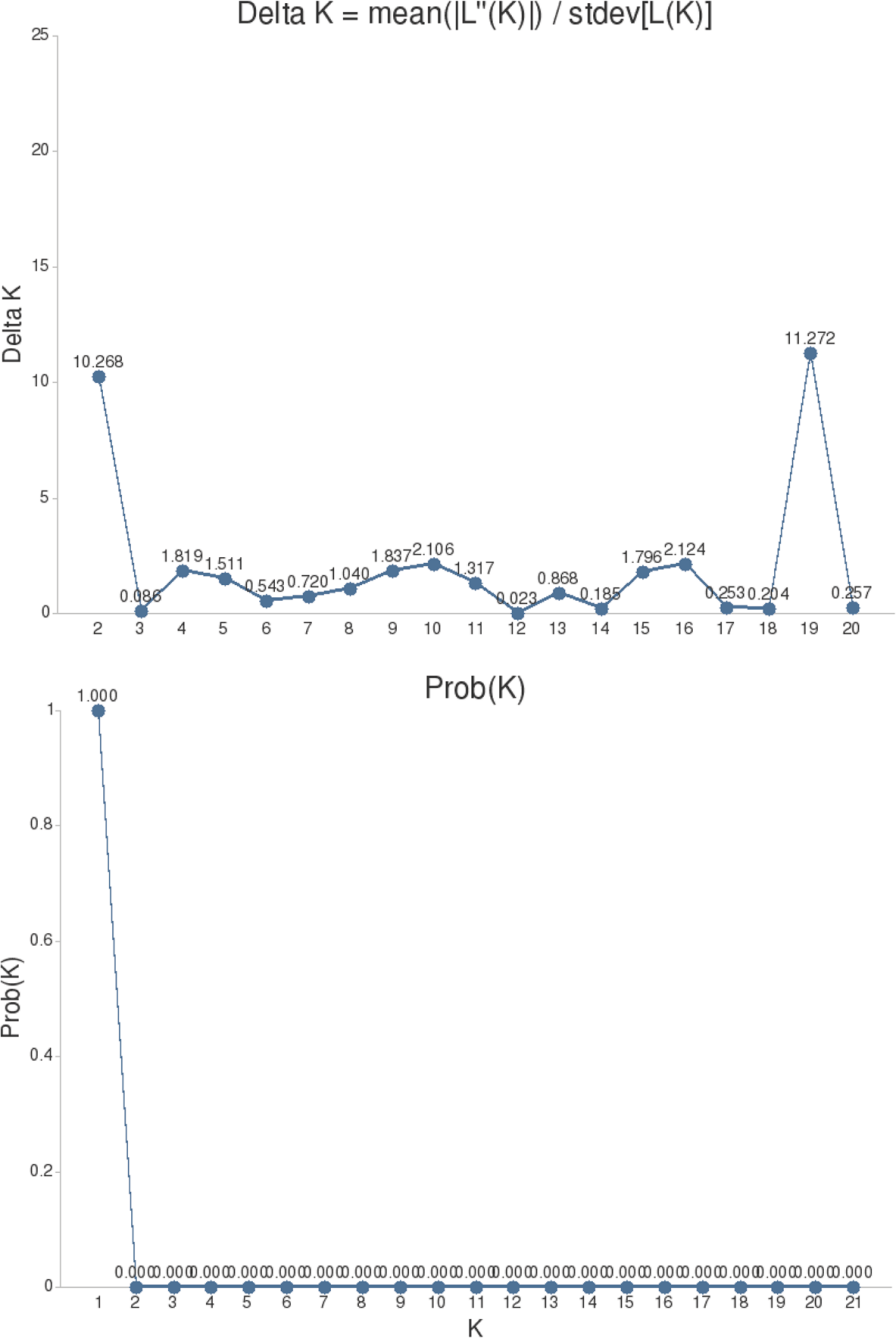
Genetic groups identified by STRUCTURE analyses

Comparing all 21 populations, ΔK as a function of K reached an initial peak at K = 2 and then reached a higher peak at K = 19 (Fig. 5). Therefore, the best value for the number of genetic clusters was 19 based on the ΔK values. However, all the individuals had very low probabilities of the assignment of a specific group under both K = 2 and K = 19 (Additional file 2: Figure S1). Furthermore, none of the closely distributed individuals showed a unique affinity to a specific group. Interestingly, and in contrast, the optimal K value determined from structure analysis was 1 based on ln(Pr(X|K)) values (Fig. 5).

**Fig. 6:**
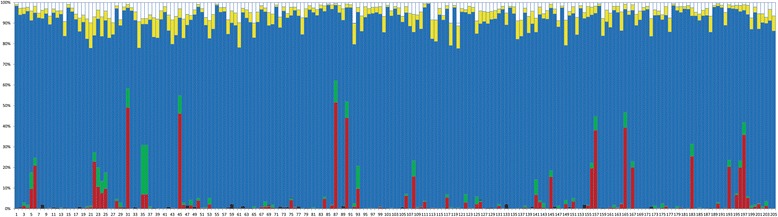
Posterior probabilities of the assignment of individuals to the following genotypic classes identified by Newhybrids analyses: red and green = parental; black = F1 hybrid; blue = F2 hybrid; and white and yellow = backcross hybrid. Each bar represents a sampled individual

The Newhybrids analyses identified almost all the individuals as F2 hybrids. One hundred six individuals had high posterior probabilities (PP > 90%), and only 6 individuals had posterior probabilities less than 50%. The highest posterior probability of individuals being one of the putative parental classes was 51%, and only 5 individuals had posterior probabilities higher than 40%. The posterior probabilities of individuals being F1 hybrids and belonging to backcross categories were less than 2% (Fig. 6).

## Discussion

The genetic divergence within the black-throated tit was noted several years ago [20, 21]. The black-throated tit has been suggested to be an unresolved species swarm, which may contain several reproductively isolated lineages given substantially paralleled divergence in morphology among its geographical populations [20]. However, because of difficulty obtaining sufficient samples, a full understanding of the genetic structure and evolutionary history within this species has not yet been achieved. Deep genetic divergence within a species warrants further investigation, particularly for a species without conspicuous morphological changes, because diverged lineages may indicate incipient speciation or completely reproductively isolated species and may provide particular insight into the drivers of speciation.

In this study, we conducted genetic analyses for populations sampled at the contact zone and hence can describe a clear pattern of genetic structure and its implication for the mitochondrial divergence within the two morphologically similar subspecies of the black-throated tit, *A. C. concinnus* and *A. c. talifuensis*. In [21], the authors found little mitochondrial genetic change between the subspecies *A. C. concinnus* and *A. c. talifuensis*. However, significant divergence was detected in another study [22]. The current study can explain this discordance. Owing to our dense sampling, we found that the deep mitochondrial break actually occurred within the subspecies *A. c. talifuensis*. The samples collected in this study belong to subspecies *A. c. talifuensis*, and phylogenetic analyses based on mitochondrial ND2 sequences clearly indicated high divergence in mitochondrial locus, as previously observed. According to the phylogeny, the haplotypes of two lineages were sympatrically distributed in 11 populations, and lineages A and B-C were found to be allopatric in 3 and 7 populations, respectively.

However, analyses of the genotype data from 11 microsatellite loci showed that the sampled populations were not significantly diverged. According to the divergence of mitochondrial data, the analyses of molecular variance for microsatellite data revealed that most genetic variation occurred within sampled populations, whereas genetic variations were small among mitochondrial-defined lineages and among sampled populations within lineages, indicating little divergence between lineages (Table 3). The genetic differentiation among sampled populations was more likely due to the isolation caused by geographical distance at this contact zone. The absence of genetic structure was further supported by the results from the analyses of structure. Although the best estimate for the number of genetic clusters was 19 based on ΔK values, this value has little biological meaning for the lower probabilities for individuals assigned to a specific genetic cluster. Consequently, the value K = 2 inferred from ΔK values should be ruled out for the same reason (Additional file 2: Figure S1). Based on ln(Pr(X|K)) values, a lack of divergence can be concluded, as the optimal K determined was one.

The discordant genetic pattern between mitochondrial and microsatellite data is in agreement with the results of a previous study [23], in which the population structure was reconstructed by using several nuclear sequence loci. By conducting coalescent analyses, this discordance was explained by male-biased gene flow, not by incomplete lineage sorting in nuclear loci [23]. However, it was unresolved whether male-biased gene flow was caused by the reproductive barrier (female hybrid sterility) that emerged and thus indicated an incipient speciation. Analyses conducted using Newhybrids can provide sufficient information to evaluate the relationship between the two deeply diverged mitochondrially defined lineages. As Newhybrids identified most of the individuals sampled as F2 hybrids with high posterior probabilities and lower posterior probabilities for the assignments of individuals to the rest of the classes, it can be concluded that diverged mitochondrial populations actually undergo random interbreeding and most likely formed a unimodal hybrid zone at this contact zone. Reproductive barriers are very weak or even absent. Therefore, the mitochondrial split is not indicative of cryptic species but most likely represents high genetic diversity within a species.

It is highly likely that the divergent mitochondrial lineages reflect a situation of “speciation in reverse” in the black-throated tit. As described by a previous study, the two targeted subspecies of the black-throated tit have been affected by Quaternary climatic oscillations [22]. Remnant populations survived in the isolated refugia and came into secondary contact when the climate became habitable. Neutral or adaptive mutations were accelerated during the time of allopatry but not to an extent that resulted in the development of prezygotic and postzygotic reproductive barriers. However, it is also likely that the reproductive barriers that emerged were negated by gene flow after contact. The high intraspecific divergence in mitochondrial loci without reproductive isolation has been reported for a handful of species, including species of birds, such as the common redstart [8] and the common raven [9].

It has been suggested that the ecology of a species [9, 60], as well as its signal traits, such as vocalizations and plumage, contributes to the likelihood of allopatric speciation [61, 62]. The allopatric lineages of generalist species have a higher probability than those of ecological specialists to remerge after secondary contact because wide ecological tolerances might decrease the probability of unique adaptations [9]. The black-throated tit is a generalist species, distributed in a variety of habitats across a wide range of altitudes, including natural shrubs, coniferous and pine-dominated forests, as well as in human-dominated landscapes, such as cities and agricultural fields. Therefore, the wide range of ecological tolerance typical of the black-throated tit might play a role in the remerging of divergent lineages without heading towards allopatric speciation. Furthermore, in accord with the hypothesis suggested by [63], historically allopatric isolation appears to have had little effect on the development of morphological differences in the black-throated tit. Differences in plumage between the two lineages (or two subspecies) are inconspicuous, with both having a reddish crown, red flank, buff belly and chestnut breast band. This is also the case for their vocalizations. A line of convincing evidence in this respect is that samples were collected by the playback of vocalizations of the black-throated tit from Taiwan in this study. Additionally, the calls documented to date were shown to be surprising similar, even among the species in genus *Aegithalos* [21], and thus much more common among the black-throated tit. Due to the lack of divergence in these traits, lineages might be less likely to evolve prezygotic reproductive barriers.

However, given the phylogeny constructed with all the available ND2 sequences from the black-throated tit, other explanations for deep divergent mitochondrial lineages within a species cannot be ruled out. The phylogenetic relationship for the targeted lineages appears to be that of a non-sister group, despite the divergence occurring within a subspecies. When including the available GenBank sequences from subspecies *iredalei,* the lineage from *iredalei* is closely related to lineage B-C, which was restricted in nominated subspecies and occupied certain individuals in *talifuensis*, whereas lineage A is the sister group to lineage B-C and *iredalei*. However, as described in the results, adding sequences from subspecies *manipurensis* significantly changed the phylogeny. *A. C. manipurensis* was the sister to lineage A, but phylogenetic relationships were unresolved among lineage B-C, *iredalei* and lineage A-*manipurensis*. Using the available GenBank Cyt B data, the phylogeny also indicated that lineage A was closely related to *manipurensis* and the sister group *iredalei*. Lineage B-C was the sister group to all three lineages. All nodes had high support in both the BI and ML trees. The non-sister relationship between the two lineages highlighted that other explanations, such as mitochondrial introgression caused by hybridization [64, 65] and the coexistence of ancestral lineages in a panmictic population [9, 66], must be taken into consideration. Without data covering the other subspecies and its relative species, these hypotheses are difficult to evaluate and thus should be the subject of future work.

## Conclusions

This study found that the divergent mitochondrial lineages of the black-throated tit formed a unimodal zone because of the absence of certain types of solid reproductive barriers. The current study has several significant implications. First, male-biased gene flow has been suggested as the driving factor for the cyto-nuclear discordance between the subspecies *A. C. concinnus* and *A. c. talifuensis* [23]. However, the male-biased gene flow can be attributed to male-biased dispersal or female hybrid sterility [67, 68], the latter of which indicates the emergence of reproductive isolation. However, Newhybrids analyses showed that almost all individuals sampled were F2 hybrids, indicating that the birds could be randomly interbreeding in the contact zone. Reproductive isolation was nonexistent, or very weak at the very least, indicating that female hybrid sterility scarcely occurred in the targeted populations. Hence, male-biased dispersal may be the main driver, which is not common in avian species. The dispersal pattern in the black-throated tit will be further addressed in our future studies. Second, the evolutionary history of the black-throated tit must be further studied because of the dramatic changes in phylogeny relative to that reported in [21] when available GenBank Cyt B sequences from four subspecies were used. More samples are needed to gain a full understanding of the phylogeny and phylogeography of the black-throated tit. Third, our results are in accord with the proposed relationship between divergence time and reproductive isolation based on the scattered studies on anurans [37, 38]. We suggest that the black-throated tit may be an excellent species for further evaluating this rule, as this species of bird contains divergent lineages with both small and substantial morphological divergences. Finally, we suggest that the subspecies *A. C. concinnus* and *A. c. talifuensis* might be better merged together, at the very least within mainland China, owing to little divergence in morphology and deep mitochondrial divergence occurring within subspecies *talifuensis* but without reproductive barriers. The nominated race has been identified based on a greater degree of red coloring than the subspecies *talifuensis* [32]. However, throughout the wide sampling conducted in this study, we found that this minor color difference actually existed within the nominated subspecies, particularly between summers and winters.

## Additional files

Additional file 1: Table S1. Sampling locations, sample size, geographic information and mitochondrial divergence of the black-throated tit for this study. (DOCX 18 kb)

Additional file 2: Fig. S1. Plots of the best K identified by the method of ΔK. (DOCX 163 kb)

Additional file 3: Microsatellite data file used for analyses. (XLSX 40 kb)

## Abbreviations

BI: Bayesian inference
Ma: Million years ago
ML: Maximum likelihood
