# Supplementary material for: The absence of reproductive isolation between non-sister and deeply diverged mitochondrial lineages of the black-throated tit (Aegithalos concinnus) revealed by a multilocus genetic analysis in a contact zone
Source: BMC Evol Biol. 2017 Dec 20;17:266. doi: 10.1186/s12862-017-1114-9 (PMC5738821; doi:10.1186/s12862-017-1114-9)
Supplement: Supplementary file 1 — Sampling locations, sample size, geographic information and mitochondrial divergence of the black-throated tit for this study. (DOCX 18 kb) [file 12862_2017_1114_MOESM1_ESM.docx]

Table S1. Sampling locations, sample size, geographic information and mitochondrial divergence of the black-throated tit for this study.

| Location | Sample size | longitude | latitude | Mitochondrial divergence | |
| --- | --- | --- | --- | --- | --- |
|  |  |  |  | Lineage A | Lineage B-C |
| Yushe | 9 | 104.7788925 | 26.4956512 | 0 | 9 |
| Faer | 10 | 104.7488403 | 26.2988016 | 3 | 7 |
| Boguo | 10 | 104.497612 | 26.0187168 | 1 | 9 |
| Fuyuan | 10 | 104.281601 | 25.6925125 | 5 | 5 |
| Weishe | 10 | 104.7365699 | 25.2880249 | 0 | 9 |
| Luoping | 2 | 104.2994385 | 24.9011803 | 0 | 2 |
| Shizhong | 20 | 103.9799881 | 24.835453 | 10 | 10 |
| Shilin | 10 | 103.2321771 | 24.792963 | 8 | 2 |
| Mile | 10 | 103.3804703 | 24.3721752 | 8 | 2 |
| Zhongheying | 9 | 103.5918503 | 23.7616425 | 1 | 8 |
| Wenshan | 10 | 104.195816 | 23.3512947 | 0 | 10 |
| Qiube | 7 | 104.2120285 | 24.0399704 | 0 | 7 |
| Songming | 10 | 103.0296478 | 25.3550034 | 7 | 3 |
| Gongshan | 10 | 103.2375259 | 25.747963 | 5 | 5 |
| Daibu | 10 | 103.4055634 | 26.2018166 | 0 | 10 |
| Kuming | 9 | 102.8334503 | 25.0878658 | 9 | 0 |
| Gejiu | 5 | 103.1386414 | 23.3977318 | 5 | 0 |
| Pingbing | 5 | 103.6687393 | 22.9878082 | 0 | 5 |
| Qujing | 10 | 103.7739792 | 25.528265 | 6 | 4 |
| Xuanwei | 19 | 104.0725861 | 26.170332 | 4 | 15 |
| Tonghai | 10 | 102.7408981 | 24.1126022 | 10 | 0 |
