# Supplementary figures and images for: The absence of reproductive isolation between non-sister and deeply diverged mitochondrial lineages of the black-throated tit (Aegithalos concinnus) revealed by a multilocus genetic analysis in a contact zone
Source: BMC Evol Biol. 2017 Dec 20;17:266. doi: 10.1186/s12862-017-1114-9 (PMC5738821; doi:10.1186/s12862-017-1114-9)

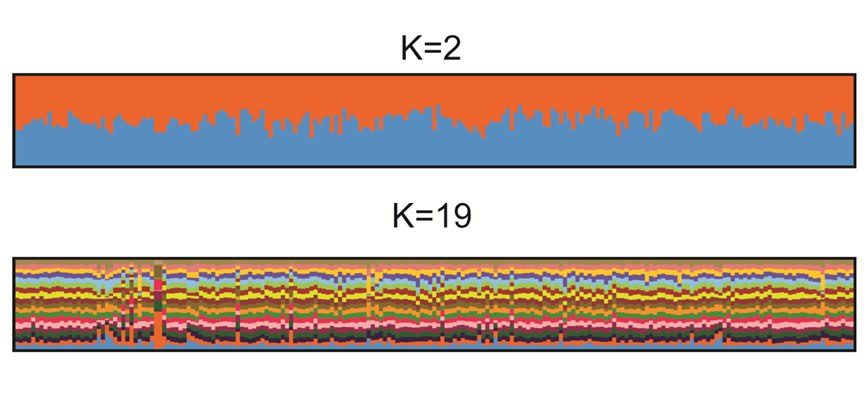


Figure S1. Plots of the best K identified by the method of ΔK.

Supplement: Supplementary file 2 — Plots of the best K identified by the method of ΔK. (DOCX 163 kb) [file 12862_2017_1114_MOESM2_ESM.docx]
